# Supplementary material for: ChromoTrace: Computational reconstruction of 3D chromosome configurations for super-resolution microscopy
Source: PLoS Comput Biol. 2018 Mar 9;14(3):e1006002. doi: 10.1371/journal.pcbi.1006002 (PMC5862484; doi:10.1371/journal.pcbi.1006002)
Supplement: S1 Text — A description of the methods used to simulate localization profiles produced by SRM along with the basic processing used to create the input files for Chromotrace. (PDF) [file pcbi.1006002.s001.pdf]

1 Supplementary Information

1.1 Simulation of Localization Event Profiles

To simulate *localization event* (LE) profiles were used our previously generated simulations as a base and converted these into a simulated LE profiles with realistic amounts of error. Only those simulations with 10 colors were used to ensure the number of colors is realistic and each of these 10 colors are encoded using a unique docking strand. First we convert the distances in simulations to nanometres which with our grid size and the $10\mu m$ diameter of the human nucleus gives us a spacing of $\approx 67nm$ between points. To simulate the genomic labeling of each locus they each consist of 10 FISH probes equally spaced across the locus. To simulate the errors found in real super resolution microscopy we additionally use the following properties.

- Each probe is given a probability of 0.3 that it failed.
- For each probe it is taken into account that we would see it several times as a number of LE's. This is modelled by drawing the number of LE's for each probe from a poisson distribution with a mean of 5.
- To simulate a realistic localization precision, for each of the LE's we determine the detected position by adding to the true position of the probe a random number in each directions drawn from a normal distribution with a sigma according to the realistic localization precisions $5nm$, $5nm$, $15nm$ in the x, y and z directions respectively.

After the above process we have a simulation of the raw output of a super resolution microscope using experimentally verified estimates for the localization precision and probe binding probability.

1.2 Processing of Localization Event Profiles

We analysed our simulated images to quantify the errors that this process introduced compared with our original simulations. Below is a summary table showing the proportion of loci and their number of successful probes.

# of probes	0	1-2	3-4	5-6	7-8	9-10
% of loci	3.5%	16.6%	31.8%	30.6%	14.7%	2.8%

Table 1: Proportion of loci with a number of successful probes

The fewer the number of successful probes in a loci, the less likely it is that we will be able to accurately locate the true centre of the loci. From the above we can see that 51.9% of the loci have 4 or fewer successful probes and 97.2% have

fewer than 9 successful probes, suggesting that this process has added a lot of variation into the simulations. To process the simulated LE profiles we take the LE information and cluster the LE's using a well know clustering algorithm called DBSCAN (Density-based spatial clustering of applications with noise). DBSCAN attempts to group together points that are in a *dense* region and mark as *outliers*, or noise, those points which are in not in dense regions. A dense region within the input data is defined by two user defined parameters: MinPts and eps. eps defines a radius around a point p and any other points within this radius are considered to be adjacent to p. MinPts defines what is considered to be a dense region in the dataset. If a point p has MinPts points (including itself) that are adjacent to it then it is in a dense region. A cluster is then defined by taking a point in a dense region and adding to the cluster this point and all reachable points that are also in dense regions. Finally any node which is adjacent to any node in the cluster is also added. For each cluster we average the x, y and z coordinates to get the final co-ordinates and this forms the input for chromotrace. For our simulations we use the default value of MinPts $= 5$ and set eps $= 15$ chosen due to the localistion precision.

We use DBSCAN as an out of the box solution and do not make any attempt to refine the clusters that are defined or to implement a more sophisticated clustering procedure. We do this to create a challenging simulation for our algorithm, although a more sophisticated clustering algorithm or a custom algorithm would improve accuracy of the localization.

After the segmentation process we again investigate the amount of error introduced by this process which can be seen in **Fig 9**. The percent of missing loci is approximately 6% both for genomes and for chromosomes (**Fig 9B**). The percentage of LE's that were clustered into the wrong locus is approximately 5.8% for both genomes and chromosomes (**Fig 9C**). The percentage of clusters that contained LE's from multiple loci and observe a mean percentage of approximately 1.9% for genomes and chromosomes (**Fig 9D**). Finally we looked at the number of loci which occur multple times in the output form our segmentation algorithm. This can occur if some of the probes are missing and DBSCAN then defines two clusters for the same loci. This type of misclassification occurs for approximately 1.5% of the loci.
